# Using machine learning to identify features associated with different types of self-injurious behaviors in autistic youth

**DOI:** 10.1017/S0033291725000637

**Published:** 2025-03-31

**Authors:** Ligia Antezana, Caitlin M. Conner, Safaa Eldeeb, Samuel Turecki, Matthew Siegel, Helmet T. Karim, Carla A. Mazefsky

**Affiliations:** 1 Department of Psychiatry, University of Pittsburgh School of Medicine, Pittsburgh, PA, USA; 2 Division of Child and Adolescent Psychiatry, Cincinnati Children’s Hospital Medical Center, Cincinnati, OH, USA; 3 Departments of Psychiatry and Pediatrics, Tufts University School of Medicine, Medford, MA, USA; 4 Department of Bioengineering, University of Pittsburgh, Pittsburgh, PA, USA

**Keywords:** autism, emotion dysregulation, medical problems, psychiatric symptoms, self-injurious behavior, repetitive behavior, machine learning, elastic net regression, ADHD, adaptive behavior, headaches, sleep, pica, inpatient, autism inpatient collection

## Abstract

**Background:**

Self-injurious behaviors (SIB) are common in autistic people. SIB is mainly studied as a broad category, rather than by specific SIB types. We aimed to determine associations of distinct SIB types with common psychiatric, emotional, medical, and socio-demographic factors.

**Methods:**

Participants included 323 autistic youth (~50% non−/minimally-speaking) with high-confidence autism diagnoses ages 4–21 years. Data were collected by the Autism Inpatient Collection during admission to a specialized psychiatric inpatient unit (www.sfari.org/resource/autism-inpatient-collection/). Caregivers completed questionnaires about their child, including SIB type and severity. The youth completed assessments with clinicians. Elastic net regressions identified associations between SIB types and factors.

**Results:**

No single factor relates to all SIB types. SIB types have unique sets of associations. Consistent with previous work, more repetitive motor movements and lower adaptive skills are associated with most types of SIB; female sex is associated with hair/skin pulling and self-rubbing/scratching. More attention-deficit/hyperactivity disorder symptoms are associated with self-rubbing/scratching, skin picking, hair/skin pulling, and inserts finger/object. Inserts finger/object has the most medical condition associations. Self-hitting against surface/object has the most emotion dysregulation associations.

**Conclusions:**

Specific SIB types have unique sets of associations. Future work can develop clinical likelihood scores for specific SIB types in inpatient settings, which can be tested with large community samples. Current approaches for SIB focus on the behavior functions, but there is an opportunity to further develop interventions by considering the specific SIB type in assessment and treatment. Identifying factors associated with specific SIB types may aid with screening, prevention, and treatment of these often-impairing behaviors.

## Introduction

Self-injurious behavior (SIB) is self-inflicted physical harm, which affects about 42% of individuals diagnosed with autism spectrum disorder (Baghdadli, Pascal, Grisi, & Aussilloux, 2003; Soke et al., 2017; Steenfeldt-Kristensen, Jones, & Richards, 2020), which is characterized by social communication differences and restricted and repetitive behaviors (American Psychiatric Association, 2013). It is imperative to better understand SIB in autistic people,^1^ as SIB is one of the most common reasons for psychiatric hospitalization (Mandell, 2008; Siegel et al., 2012), and is linked to increased caregiver stress, diminished family well-being (Tint & Weiss, 2016), and social isolation, including limitations on education and residential opportunities (Cantin-Garside et al., 2021; Park et al., 2009). SIB ranges in severity from minor injuries to severe tissue damage and spans a wide range of types including but not limited to: self-hitting, self-biting, skin picking, hair-pulling, eye-gouging, and self-rubbing/scratching (Bodfish, Symons, Parker, & Lewis, 2000). The impact of SIB on the person’s health (i.e., tissue damage, eyesight loss, and concussions) is a serious concern (Hyman, Fisher, Mercugliano, & Cataldo, 1990), especially since SIB commonly persists into adulthood (Laverty et al., 2020). Despite the often debilitating impact and high prevalence, progress on interventions for SIB has been at a relative standstill for decades, with the gold standard approach being functional behavior analysis (Iwata et al., 1982; Kahng, Iwata, & Lewin, 2002; Shawler et al., 2019). One limiting factor in SIB research is that different types of SIB are typically grouped together. Parsing the heterogeneity of SIB into distinct types may improve the screening, identification, and treatment of SIB. Thus, we aimed to identify theory-driven factors that may have associations with distinct SIB types in a well-characterized sample of autistic youth admitted to specialized inpatient psychiatric settings, utilizing a data-driven machine learning approach.

The taxonomy of SIB in autistic samples is not well understood or agreed upon (Steenfeldt-Kristensen, Jones, & Richards, 2020; Turner, 1999). Definitions vary, with some highlighting that SIB are often ‘highly repetitive and rhythmic’ and different from intentional behaviors such as suicidal behaviors, non-suicidal self-injury, or other self-harm behaviors (Fee & Matson, 1992; Weiss, 2003). The classification and taxonomy of SIB in autistic samples require further study, especially given the emerging work on high rates of suicidality and non-suicidal self-injury in autistic people (e.g., Cassidy et al., 2014; Conner et al., 2020; Maddox, Trubanova, & White, 2017; Moseley et al., 2019). One common measure of SIB in autistic samples is the SIB subscale of the Repetitive Behavior Scale-Revised (RBS-R; Bodfish, Symons, Parker, & Lewis, 2000), which measures the severity (frequency, how difficult it is to interrupt, and interference) of eight common SIB types: hitting self with body part, hitting self against surface or object, hitting self with object, biting self, pulling hair or skin, self-rubbing or scratching, inserts finger or object (e.g., eye-poking and ear-poking), and skin picking. Some SIB measures include other behaviors that are often conceptualized as broader self-harm, like pica, vomiting, and teeth grinding (Rojahn et al., 2012); such behaviors are excluded from our definition and measurement here. As the definition of SIB in autistic samples has primarily emerged from intellectual and developmental disabilities, the theories of SIB in autistic people have primarily focused on functional behavioral approaches for the study of SIB (Iwata et al., 1982; Shawler et al., 2019).

These functional behavioral theories highlight how SIB emerges and is maintained in relation to environmental reinforcements (e.g., access to tangibles, social attention, automatic, and escape/avoidance; Iwata et al., 1982; Shawler et al., 2019). In contrast, conceptualizations of self-injury in non-autistic people focus on physical and emotional pain (Brown, Quetsch, Aloia, & Kanne, 2024; Chapman, Gratz, & Brown, 2006; Hooley & Franklin, 2018; Kao et al., 2024). For example, SIB may be used to regulate physical pain (e.g., distraction) and emotions (e.g., up-regulate low-intensity emotions and down-regulate high-intensity emotions). Due to the focus on behavioral function in the autism SIB literature, there is a paucity of research on other important conceptualizations that may aid our understanding of SIB and the development of targeted interventions. In the domain of physical pain, there is some suggestion of altered pain systems in autistic samples (Sandman, 1990), but there are also frequent findings of links between painful or uncomfortable medical conditions and SIB (Bosch, Van Dyke, Smith, & Poulton, 1997; de Winter, Jansen, & Evenhuis, 2011). In autistic child samples, more SIB is associated with more pain-related behaviors (Courtemanche, Black, & Reese, 2016; Richards, Davies, & Oliver, 2017), and the presence of medical conditions, including stomach problems, visual impairments, and oral pain (de Winter, Jansen, & Evenhuis, 2011; Folch et al., 2018; Neuhaus, Bernier, Tham, & Webb, 2018).

There is also limited work on emotions and their relationship to SIB in autistic people. There are important links that have been found between co-occurring psychiatric symptoms and SIB. Both internalizing (i.e., anxiety and depression) and externalizing symptoms (i.e., overactivity, impulsivity, and aggression) are associated with increased SIB in autistic children and adults (Jasim & Perry, 2023; Muskett et al., 2019; Richards, Moss, Nelson, & Oliver, 2016; Soke et al., 2017; Vandewalle & Melia, 2021). Notably, in autistic children with severe SIB, attention-deficit/hyperactivity disorder (ADHD) is commonly co-occurring (66.7%; Fong et al., 2024). Greater irritability, aggression, disruptive behavior, and anger dysregulation are also linked to SIB (Brown, Quetsch, Aloia, & Kanne, 2024; Flowers, Lantz, Hamlin, & Simeonsson, 2020; Soke et al., 2017).

An important factor that underlies many of the aforementioned psychiatric factors is emotion dysregulation, which is defined as difficulty in altering one’s own emotions in a goal-directed manner (Gross & Thompson, 2007; Mazefsky et al., 2013). Two areas of emotion dysregulation are: (1) reactivity (intense, rapidly escalating, and sustained emotional reactions), and (2) dysphoria (states of unease, sadness, and low positive affect; Mazefsky, Day, et al., 2018a; Mazefsky, Yu, et al., 2018). Intriguingly, emerging work has uncovered significant links between emotion dysregulation and SIB in autistic people; however, this critical connection remains understudied (Goldfarb et al., 2021; Martínez-González, Cervin, & Piqueras, 2021; Northrup et al., 2022; Samson et al., 2014). Linkages between distinct types of SIB and emotion dysregulation have not been examined, and reactive and dysphoric presentations may differentially relate to specific SIB types.

Finally, certain demographic variables, such as age, sex assigned at birth, and socioeconomic status relate to overall SIB in autistic individuals. Younger autistic children may display more SIB (Esbensen, Seltzer, Lam, & Bodfish, 2009), and overall SIB may be more prevalent in autistic females than males (Beggiato et al., 2016; Cohen et al., 2010; Neuhaus, Webb, & Bernier, 2019). Autistic girls demonstrate more hair/skin pulling and self-rubbing/scratching, as compared to autistic boys (Antezana et al., 2019). Regarding socioeconomic status factors, families of autistic children with SIB are more likely to have lower household income, lower maternal education, and greater reliance on public health insurance (Soke et al., 2016, 2017). However, these associations between SIB and demographic variables are not consistently found across studies, and may vary based on sample characteristics.

Due to the need to make advances in this area and the field’s general focus on overall SIB, we examined variables that may be associated with distinct SIB types, utilizing a well-characterized sample of autistic youth admitted to a specialized inpatient setting, an ideal sample due to high rates of SIB as well as significant variability in potential variables of interest (i.e., speaking ability and emotion dysregulation). We aimed to leverage a machine learning approach, elastic net regressions with cross-validation, to assess the relationship between types of SIB and various factors, including medical conditions, psychiatric symptoms, emotion dysregulation, cognitive and adaptive abilities, behaviors associated with autism, and demographics. This method was chosen for its ability to identify robust features associated with the various SIB types, even in the presence of high multicollinearity in the data. Although this study was primarily discovery-based, we chose variables based on themes previously identified in the literature. We hypothesized that the statistical models would differ across the various SIB types.

## Method

### Participants and procedures

The Autism Inpatient Collection (AIC) is a multi-site study of specialized inpatient psychiatric units for youth with autism spectrum disorder and other developmental disorders. The full methods of the AIC have been published previously (Siegel et al., 2015). Participants with a Social Communication Questionnaire-Lifetime Version (SCQ; Rutter, Bailey, & Lord, 2003) score above the clinical cutoff (≥12), or with high suspicion of autism from the inpatient team were eligible for enrollment. Inclusion in the AIC dataset required confirmation of autism spectrum disorder diagnosis by research-reliable administration of the Autism Diagnostic Observation Schedule-2 (ADOS-2; Lord et al., 2012) and meeting DSM-5 criteria. Exclusion criteria included the lack of availability of a caregiver proficient in English or the prisoner status of the autistic individual. The present study is a secondary analysis that utilizes admission data from Phase I of the AIC (collected between 2013 and 2017), and includes participants with caregiver report data for the Repetitive Behavior Scale-Revised (RBS-R; Bodfish, Symons, Parker, & Lewis, 2000) SIB items (*N* = 323; Table 1).

**Table 1.** Participant demographics and characteristics

	*n*	%
Sex assigned at birth		
Female	68	21.05%
Male	255	78.95%
Race		
Asian	8	2.48%
Black	32	9.91%
Native American	3	0.93%
White	252	78.02%
Multiracial	19	5.88%
Other	3	0.93%
Missing	6	1.86%
Ethnicity		
Hispanic/Latino	20	6.19%
Not Hispanic/Latino	289	89.47%
Missing	14	4.33%
Nonspeaking or minimally speaking (ADOS–2 Module 1 or 2)	163	50.46%
Parental education		
Less than 8th grade	2	0.6%
Some high school	12	3.7%
Finished high school (or equivalent)	60	18.6%
Some college or associate (AA) degree	114	35.3%
Bachelor’s degree (BA, BS)	60	18.6%
Post-graduate degree	54	16.7%
Missing	21	6.5%
Household income		
Less than $20,000	56	17.3%
$21,000 to $35,000	59	18.3%
$36,000 to $50,000	40	12.4%
$51,000 to $65,000	32	9.9%
$66,000 to $80,000	17	5.3%
$81,000 to $100,000	21	6.5%
$101,000 to $130,000	26	8.0%
$131,000 to $160,000	14	4.3%
Over $160,000	20	6.2%
Missing	38	11.8%

	M (SD)	Range
Age (years)	12.91 (3.37)	4.10–21
Leiter–3 Nonverbal IQ Standard Score	75.05 (28.67)	30–145
ADOS–2 Comparison Score	7.79 (1.59)	4–10
EDI Dysphoria Theta Score	.47 (.80)	−1.36–2.77
EDI Reactivity Theta Score	.83 (.79)	−1.99–2.11
RBS-R 1 ‘Hits self with body part’ Rating	1.43 (1.18)	0–3
RBS-R 2 ‘Hits self against surface or object’ Rating	1.35 (1.19)	0–3
RBS-R 3 ‘Hits self with object’ Rating	0.76 (1.02)	0–3
RBS-R 4 ‘Bites self’ Rating	0.94 (1.18)	0–3
RBS-R 5 ‘Pulls’ Rating	0.80 (1.07)	0–3
RBS-R 6 ‘Rubs or scratches self’ Rating	0.97 (1.11)	0–3
RBS-R 7 ‘Inserts finger or object’ Rating	0.42 (0.79)	0–3
RBS-R 8 ‘Skin picking’ Rating	1.01 (1.13)	0–3

## Measures

### Self-injurious behavior

Caregivers completed the RBS-R SIB (Bodfish, Symons, Parker, & Lewis, 2000) measure for their child, which consists of 8 distinct SIB types (Table 2). The instructions note that when scoring one should consider the behavior frequency, how difficult it is to interrupt, and how much it interferes. Higher scores indicate more severe SIB. Each item was rated on a 4-point scale ranging from 0 ‘Behavior does not occur’ to 3 ‘Behavior occurs and is a severe problem.’ The full range of item level (distinct SIB type) rating scores were used as dependent variables in the analyses. In our sample, the internal consistency of this measure was acceptable (Cronbach’s ⍺ = .70). Generally, all items were positively and highly correlated with each other (*r*s between .26 and .65, *p*s < .001), though ‘skin picking’ had lower strengths in relationships with ‘hits self against surface or object’ (*r* = .11, *p* < .05) and ‘bites self’ (*r* = .17, *p* < .01).

**Table 2.** Results of elastic net regression for the RBS-R SIB items

Out-of-bag prediction accuracy	*r* = 0.42, *p* < 0.001, α = 0, λ = 2.57	*r* = 0.36, *p* < 0.001, α = 0, λ = 5.26	*r* = 0.23, *p* < 0.001, α = 0, λ = 4.44	*r* = 0.30, *p* < 0.001, α = 0, λ = 3.22	*r* = 0.26, *p* < 0.001, α = 0, λ = 4.56	*r* = 0.30, *p* < 0.001, α = 0, λ = 5.96	*r* = 0.28, *p* < 0.001, α = 0, λ = 2.86	*r* = 0.20, *p* < 0.005, α = 0, λ = 3.62
	Hits self with body part (hits or slaps head, face, or other body area)	Hits self against surface or object (hits or bangs head or other body part on table, floor, or other surface)	Hits self with object (hits or bangs head or other body area with objects)	Bites self (bites hand, wrist, arm, lips or tongue)	Pulls (pulls hair or skin)	Rubs or scratches self (rubs or scratches marks on arms leg, face, or torso)	Inserts finger or object (eye-poking, ear-poking)	Skin picking (picks at skin on the face, hands, arms, legs or torso)
Predictors	RBS-R 1 (ß)	RBS-R 2 (ß)	RBS-R 3 (ß)	RBS-R 4 (ß)	RBS-R 5 (ß)	RBS-R 6 (ß)	RBS-R 7 (ß)	RBS-R 8 (ß)
**Autism-related measures**								
Repetitive motor movements (ABC Stereotypy Subscale)	0.107	0.046	0.033	0.045	0.04	0.031	0.033	
Inappropriate Speech (ABC Inappropriate Speech Subscale)				0.033			0.018	0.032
Lifetime Level of Autistic Traits (SCQ Total Score)	0.043				0.022			
Clinician-Rated Level of Autistic Traits (ADOS–2 Comparison Score)				0.037			0.021	
Social Withdrawal (ABC Lethargy Subscale)			0.025					
**Demographics**								
Assigned Female at Birth (Assigned Male at Birth Reference)					0.033	0.019		
Total Household Income				−0.033				
Parental Education	−0.039							
**Cognitive and adaptive behavior measures**								
Communication skills (Vineland-II Communication Domain SS)	−0.047	−0.040	−0.017	−0.042		−0.014		
Daily living skills (Vineland-II Daily living skills Domain SS)	−0.039	−0.028		−0.035	−0.026	−0.019		
Socialization skills (Vineland-II Socialization Domain SS)	−0.037	−0.035	−0.017	−0.037				
Adaptive behavior skills (Vineland-II Adaptive Behavior Composite SS)		−0.028			−0.018			
IQ (Leiter Nonverbal IQ SS)	−0.053	−0.029		−0.047				
**Medical conditions**								
Headaches		0.023	0.020			0.017	0.021	
Allergies	0.040						0.022	0.028
Pica				0.038			0.024	
Sleep problems		0.024		0.034				
Concussion or head injury			0.032					
Dental problems						0.021		
Stomach problems or pain								0.033
High prolonged fever							0.02	
Significant accidents			0.026					
**Psychiatric symptom severity**								
ADHD Hyperactivity/Impulsivity Symptoms (CASI ADHD Hyperactive/Impulsive T-score)					0.026	0.022		0.028
ADHD Inattentive Symptoms (CASI ADHD Inattentive T-score)						0.017	0.019	
ADHD Combined Symptoms (CASI ADHD Combined T-score)						0.02		
Hyperactivity (ABC Hyperactivity Subscale)								0.03
Oppositional Defiant Symptoms (CASI Oppositional Defiant T-score)	−0.044			−0.036				
Separation Anxiety Symptoms (CASI Separation Anxiety T-score)					0.021			0.029
Schizophrenia Symptoms (CASI Schizophrenia T-score)			0.019					
**Emotion dysregulation inventory items**								
EDI 50: Becomes upset without a clear reason	0.041	0.020				0.017		
EDI 10: Destroys property on purpose			0.025					0.03
EDI 19: Has extreme or intense emotional reactions		0.021	0.021					
EDI 21: Hard to calm him/her down when he/she is mad or upset	0.046	0.023						
EDI 26: Physically attacks people		0.020		0.028				
EDI 6: Cannot calm down without help from someone else				0.031				
EDI 7: Suddenly switches to an opposite emotion		0.020						
EDI 8: Frustrates easily						0.018		
EDI 24: Reactions are so intense that they have to be removed from an activity or place		0.019						
EDI 28: When upset or angry, they stay that way for a long time							0.017	
EDI 36: Has trouble calming themselves down	0.033							
EDI 63: Seems sad or unhappy (Dysphoria)						0.02		
EDI 64: Appears uneasy through the day (Dysphoria)				0.032				

*Note:* Each RBS-R SIB item (1–8) is shown, as along with the item description. Out-of-bag prediction accuracy (using Pearson correlation, *r*) is shown with associated permuted p-value and the cross-validated model parameters (α and λ). Only predictors that were significant in at least one model are shown. The predictors are sorted by category (i.e., demographics) and the number of models they were significant for (e.g., repetitive motor movements significantly predicted every type except skin picking). We report the ß value for each significant coefficient, with all permuted p-values <.05. Out-of-bag prediction accuracy represents how well the model performed in a held-out testing set when trained on a separate training set. Negative beta weights are highlighted in shades of pink, positive beta weights are highlighted in shades of purple, and non-significant relationships are in gray.

### Demographic form

Caregivers completed a form with demographic and medical history information for their child. Medical variables included a history of asthma, allergies, concussion, stomach problems (i.e., stomach ulcers and abdominal pain), bowel and bladder problems (i.e., urinary tract infections and constipation), dental problems, ear infections, headaches, hearing loss, pica, fever, seizures, significant accidents, tics/Tourette’s, visual/eye problems, and sleeping problems. Caregivers reported autism simplex/multiplex family status, known genetic conditions, and child trauma history.

### Direct assessment measures

Participants were administered the ADOS-2, from which the ADOS-2 clinical comparison score (Lord et al., 2012) was derived to measure the level of autistic traits. Participants were also administered the Leiter-3 (Roid, Miller, Pomplun, & Koch, 2013) Nonverbal IQ test, used to measure nonverbal cognitive ability.

### Caregiver report measures

In addition to the RBS-R SIB measure and demographics form, caregivers also completed the SCQ (Rutter, Bailey, & Lord, 2003), Vineland-II (adaptive behavior; Sparrow, Cicchetti, & Balla, 2005), Aberrant Behavior Checklist (ABC; behavioral challenges; Aman, Singh, Stewart, & Field, 1985), Child and Adolescent Symptom Inventory (CASI; psychiatric symptoms; Gadow & Sprafkin, 2010), and Emotion Dysregulation Inventory (EDI; reactivity and dysphoria; Mazefskyet al., 2018a, 2018b). These measures were administered to a primary caregiver for the participant within 10 days of admission to the hospital unit. See Supplementary Materials S1 for more measure information.

## Data analysis plan

This analysis included 323 participants with a completed RBS-R SIB. All statistical analyses used R. We conducted a comprehensive examination of the missing data in our study through both descriptive statistics and data distribution analyses. Our findings revealed no significant patterns of missingness, suggesting that the missing data can be regarded as missing at random. We then conducted multiple imputations using the random forest approach in the ‘mice’ package (Doove, Van Buuren, & Dusseldorp, 2014). This method uses a random forest regression and classification to determine missing values for each variable. This is done with resampling (bootstrapping with replacement), choosing a subset of variables and generating multiple decision trees. Seventy-five independent variables and eight outcome variables were included in the imputation, as is the standard model for imputation (Moons, Donders, Stijnen, & Harrell, 2006). It is important to emphasize that, although the imputation process encompassed all variables, the outcome variables (RBS-R items) were not subject to imputation. This approach is preferred because it effectively captures important associations though no RBS-R data was missing (van Ginkel, Linting, Rippe, & van der Voort, 2020). A multiple imputation procedure was conducted with five imputations to address missing values in the dataset prior to model development. The imputed dataset was then utilized to train a cross-validated machine learning model, specifically elastic net regression.

Elastic net regression is a form of regularized regression that is well-suited for handling multi-collinearity among predictor variables. Elastic net uses a penalty term (λ) to penalize each additional non-zero ß value in the model. It uses a mixing parameter (α) that balances between two types of penalties: L1 norm (|ß|) and L2 norm (ß^2^), where when α = 1 it uses the L1 norm, and when α = 0 it uses the L2 norm. These are commonly referred to as LASSO (least absolute shrinkage and selection operator; Tibshirani, 1996) and ridge regression (Marquardt & Snee, 1975), respectively. By optimizing the value of α the elastic net can adaptively combine the strengths of both the LASSO and ridge regressions to identify an appropriate balance of coefficient shrinkage and variable selection.

We used elastic net regression with the eNetXplorer in R (Candia & Tsang, 2019). We modeled each of the eight RBS-R SIB items separately (eight cross-validated models), as they each represent a distinct type of SIB. For each model (i.e., each RBS-R SIB item), we included all of the features above to predict one of eight RBS-R items. In this context, ‘prediction’ pertains to our model’s ability to accurately identify self-injurious behavior (SIB) types, as demonstrated by its strong performance during cross-validation. For each model, we conducted 5-fold cross-validation and optimized over values of α (0–1) and λ (from a set of 50 pre-chosen values) – optimizing the Pearson’s correlation between actual RBS-R SIB items and out-of-bag predicted RBS-R SIB items (Zou & Hastie, 2005). This process was repeated over 500 runs, and then we permuted the dependent variable and conducted this process again (250 permutations) to compute null bounds on the predictive accuracy of the model and each independent variable. We used an approach that was able to compute *p*-values for the models and each independent variable (Candia & Tsang, 2019; Lockhart, Taylor, Tibshirani, & Tibshirani, 2014; Phipson & Smyth, 2010).

## Results

All models were significant for each SIB type (*p*s < .005) with the most variance explained for the SIB type ‘hits self with body part’ (17.64%, *r* = 0.42), followed by ‘hits self against surface or object’ (12.96%, *r* = 0.36), ‘bites self’ (9%, *r* = 0.30), ‘rubs or scratches self’ (9%, *r* = 0.30), ‘inserts finger or object’ (7.84%, *r* = 0.28), ‘pulls’ (6.76%, *r* = 0.26), ‘hits self with object’ (5.29%, *r* = 0.23), and ‘skin picking’ (4%, *r* = 0.20). Each SIB type had a unique set of predictors; Table 2 shows the significant results for each of the eight models. Thirty-three variables were not significant for any of the models and thus not included in Table 2 (Supplementary Materials S2).

The variable with the greatest number of SIB type associations was more repetitive motor movements (associated with 7/8 SIB types, *p*s < .001 [all types except ‘skin picking’]). Lower communication was associated with 5/8 SIB types (except ‘pulls,’ ‘inserts finger or object,’ and ‘skin picking’), and daily living skills were associated with 5/8 SIB types (except ‘hits self with an object,’ ‘inserts finger or object,’ and ‘skin picking’), *p*s < .05.

‘Hits self with body part’ was also associated with more lifetime autistic traits, lower parental education level, less socialization skills, lower IQ, history of allergies, less oppositional defiant symptoms, and higher scores on 3 EDI-Reactivity items (becomes upset without clear reason, hard to calm down when mad/upset, and has trouble calming themselves down).

‘Hits self against surface or object’ was also associated with less socialization, and adaptive behavior skills, lower IQ, history of headaches and sleep problems, and higher scores on six EDI-Reactivity items (becomes upset without clear reason, has extreme or intense emotional reactions, hard to calm down when mad/upset, physically attacks people, suddenly switches to an opposite emotion, and reactions are so intense that they had to be removed from an activity/place).

‘Hits self with object’ was also associated with social withdrawal, fewer socialization skills, histories of headaches, concussions/head injuries, and significant accidents, more schizophrenia symptoms, and higher scores on two EDI-Reactivity items (destroys property on purpose, and extreme or intense emotional reactions).

‘Bites self’ was also associated with more inappropriate speech, greater clinician-rated autistic traits, lower household income, less socialization skills, lower IQ, histories of pica and sleep problems, less oppositional defiant behaviors, higher scores on two EDI-Reactivity and 1 EDI-Dysphoria items (physically attacks people, cannot calm down without help from someone else, and appears uneasy through the day).

‘Pulls’ was also associated with more lifetime levels of autistic traits, being assigned female at birth, fewer adaptive behavior skills, more ADHD hyperactive/impulsive symptoms, and more separation anxiety symptoms.

‘Rubs or scratches self’ was also associated with being assigned female at birth, a history of headaches and dental problems, more ADHD hyperactive/impulsive, inattentive, and combined symptoms, and higher scores on two EDI-Reactivity and 1 EDI-Dysphoria items (becomes upset without a clear reason, frustrates easily, and seems sad or unhappy).

‘Inserts finger or object’ was also associated with more inappropriate speech, more clinician-rated autistic symptoms, histories of headaches, allergies, pica, and high prolonged fever, more ADHD inattentive symptoms, and higher scores on 1 EDI-Reactivity item (when upset or angry they stay that way for a long time).

‘Skin picking’ was associated with more inappropriate speech, histories of allergies and stomach problems, more ADHD hyperactive/impulsive symptoms, more hyperactivity, more separation anxiety, and higher scores on one EDI-Reactivity item (destroys property on purpose).

## Discussion

The present study used a machine learning elastic net regression approach to investigate associations between various theory-driven factors (including medical conditions, psychiatric symptoms, emotion dysregulation, cognitive and adaptive abilities, behaviors associated with autism, and demographics) and distinct SIB types in autistic youth. A greater understanding of these associations could be helpful for screening, caregiver guidance, and elucidating potential pathways for improved intervention. Although we know there are important relationships between SIB as a broad category and certain factors, we have found that those relationships may not hold for all SIB types, and there may be distinct associations with different SIB types. Consistent with previous literature, we found that more repetitive motor movements were strongly associated with 7/8 SIB types, followed by communication and daily living skills (each associated with 5/8 types). Skin-picking presented a distinct pattern of associations compared to other SIB types. Moreover, we found that a history of headaches and allergies were the medical conditions most frequently associated with SIB types. Lower cognitive ability and adaptive skills were strongly associated with hitting self against surface/object with body part and self-biting. Several psychiatric symptoms were related to SIB types. ADHD symptoms were linked with self-rubbing/scratching, skin picking, hair/skin pulling, and inserts finger/object, suggesting that inattention or impulsivity may be predispose to these forms of self-injury. We also found that the inserts finger/object type had the most medical condition associations, which may relate to the presence of physical stimuli due to the medical condition (e.g., ear pain from an ear infection). Assigned female at birth was associated with hair/skin pulling and self-rubbing/scratching presentations. A number of emotion dysregulation features were related to distinct SIB types; in particular, self-hitting against surface/object had the most emotion dysregulation associations of any SIB type.

Consistent with previous work (Barnard-Brak et al., 2015; Duerden et al., 2012; Folch et al., 2018; Hyman, Fisher, Mercugliano, & Cataldo, 1990; McClintock, Hall, & Oliver, 2003; Rattaz, Michelon, & Baghdadli, 2015), the current findings indicate that more repetitive motor movements showed significant associations with 7/8 SIB types. It may be helpful for future research on the linkage between repetitive motor movements and SIB to further examine shared difficulties in inhibition, habitual or compulsive tendencies, and altered arousal, as this may reveal new mechanisms for intervention in autistic people (Oliver, Petty, Ruddick, & Bacarese-Hamilton, 2012; Turner, 1999).

Notably, skin picking was the only SIB type *not* associated with repetitive motor movements or autism symptoms (aside from inappropriate speech). Skin picking was associated with more hyperactivity, separation anxiety, property destruction, and a history of allergies and stomach problems/pain. Considering this set of associations, skin picking may potentially be thought of as a truly distinct form of SIB in autistic youth, and possibly more founded in mechanisms related to anxiety, hyperactivity, or medical problems than repetitive motor movements. Similar to skin picking, separation anxiety symptoms were also associated with more hair/skin pulling.

Other autism-related behaviors are differentially associated with specific SIB types. For example, caregiver-reported level of lifetime autistic traits (SCQ) was associated with self-hitting with body parts and pulling, while clinician-rated level of autistic traits (coded from the ADOS-2) was associated with self-biting and inserting fingers/objects. Thus, there may be important connections between how autistic traits are measured and their associations with SIB types. For example, the SCQ asks several questions about autistic traits in early childhood, while the ADOS-2 is performed through clinician observation and coding of various behaviors during the semi-structured assessment.

Other interesting patterns emerged in relation to psychiatric symptoms. First, ADHD symptoms were positively associated with hair/skin pulling, self-rubbing/scratching, and inserts finger/object, suggesting a need for further research on the role of impulsivity or other aspects of ADHD in SIB in autism. Second, greater schizophrenia symptoms were associated with more self-hitting with objects. The relationship between self-injury and psychosis symptoms is well documented (which may occur due to distress from internal stimuli; Hielscher et al., 2018; Honings, Drukker, Groen, & Os, 2016; Lorentzen, Mors, & Kjær, 2022), but this relationship has not been previously explored in autistic people. Interestingly, less oppositional/defiant symptoms was associated with more self-hitting with body parts and self-biting, suggesting that these SIB types at high levels may not be done on purpose or defiantly. More work is needed to understand this relationship, including linkages with other externalizing behaviors such as aggression, as it may be the case that autistic youth with low levels of opposition/defiance are more likely to hit themselves than others when distressed. This relationship may be more complicated with self-biting, as both low defiance and ‘physically attacking people’ were linked to this type, and there may be important temporal relationships between these patterns (e.g., biting as a form of self-regulation during low levels of distress and high levels of distress linking with aggression). As the first line of intervention for SIB in autism typically relies on behavioral function, our findings may suggest ways to conceptualize alternative or additive interventions.

Although literature in non-autistic samples has associated emotion dysregulation with self-injury, there is limited work on autistic people on this linkage (Northrup et al., 2022). Our results linked several emotion dysregulation factors to different SIB types. For example, self-hitting against surface/object was associated with several reactivity items including intense reactions, hard to calm down, physical attacks, and becoming upset without a clear reason or suddenly switching emotions. This contrasts with self-rubbing/scratching, which is associated with getting frustrated easily and seeming sad or unhappy. Considering the strong link between non-suicidal self-injury and emotion dysregulation in general samples (Brown, Quetsch, Aloia, & Kanne, 202; Klonsky & Glenn, 2009), it is important for future work to examine SIB as a potential regulatory behavior for emotional or physical pain (Skegg, 2005; Wilhelm et al., 1999). Understanding the role of emotion dysregulation, and factors that may specifically relate to types, may add to the current behavioral approaches for treating SIB (Iwata et al., 1982). For example, a patient with self-rubbing/scratching may benefit from strategies to build frustration tolerance and cope with negative feelings, while a patient with self-hitting against surface or object may benefit from emotional awareness, relaxation, and breathing techniques.

In our sample, several medical conditions were uniquely associated with distinct SIB types, with a history of headaches and allergies being the most frequently associated variables. Inserting finger/object exhibited the greatest number of medical condition associations (history of headaches, allergies, pica, high prolonged fever), making it essential for healthcare providers to pay special attention to co-occurring medical conditions when assessing a child with inserting finger/object SIB (Bosch, Van Dyke, Smith, & Poulton, 1997; de Winter, Jansen, & Evenhuis, 2011). Moreover, because sensory differences are common in autism, parsing the influence of sensory differences and physical pain with these correlates may aid in understanding unique pathways linking medical conditions and SIB in autistic people.

Lower cognitive abilities and adaptive skills were associated with 6/8 SIB types. Interestingly, self-hitting against surface/object, with body parts, and self-biting showed the most significant associations in this category. Considering that repeated concussions from self-hitting are of concern, it will be important for future work to study these relationships in a bi-directional manner. Moreover, future research may focus on targeting communication, daily living, and socialization skills, as frustration associated with these skills may create scenarios in which these SIB types emerge.

Results also showed that three demographic variables were associated with specific SIB types. We found that female sex assigned at birth was strongly associated with more hair/skin pulling and self-rubbing/scratching. This finding is consistent with previous research with female autistic individuals presenting with more overall SIB as compared to males (Beggiato et al., 2016), and findings of more skin/hair pulling and self-rubbing/scratching by youth assigned female at birth (Antezana et al., 2019). In our sample, we found that lower household income was linked to more self-biting, while lower parental education was linked to more hitting self with body parts. Future work may focus on parsing these associations further, as these factors may be important proxies for other needs (i.e., food insecurity and access to intervention).

As this study provides robust factors that are strongly associated with distinct SIB types in autistic youth, an important future direction of this work is the development of clinical scores for SIB types in autistic youth. An obvious first step is developing a simplified scale to assess features linked to the likelihood of a SIB type presenting. For example, an autistic youth who presents with low cognitive and adaptive abilities, a history of headaches and sleep problems, and elevated scores for emotion reactivity (such as becoming upset without clear reason, extreme/intense emotional reactions, hard to calm down when mad/upset, physically attacks people, suddenly switching to an opposite emotion, and intense reactions requiring removal from an activity/place), together could indicate a high likelihood of self-hitting against object/surface. This approach may be individualized by collaborating with caregivers to understand what the behavior may look like for an individual (e.g., frequency, intensity, duration, and body part affected) and taking steps to proactively modify the environment and prepare caregivers (e.g., table edge cushions, use of a helmet, and discuss common triggers/patterns).

The present study has several strengths, including the examination of SIB at the topography (type) level in a well-characterized sample of autistic youth requiring inpatient services, an understudied population in autism research (e.g., Autism CARES Act, 2024). However, this work has a number of limitations. First, it is important to consider the generalizability of the sample, as many autistic individuals in this study had high support needs (e.g., IQ *M*[SD] = 75.05[28.67], and over half of the sample was characterized as non- or minimally-speaking). Additionally, the sample consisted of primarily white, non-Hispanic, male participants, and it will be important to examine these linkages associations in more diverse samples (i.e., community-based, outpatient samples which may generalize to all autistic people). Our current models were able to explain between 4% (skin picking) and 17.64% (self-hitting with body parts) of the variance in the SIB types. There could be several other associated variables that are not included here, or included but are biased by our sample characteristics that may require future study. Although we used predictive modeling, the data were collected at one-time point, and these associations cannot be interpreted in a causal way, thus, the work here is not able to disentangle what came first (i.e., allergies or self-rubbing/scratching), and it will be important for future work to examine causal relationships (Laverty et al., 2020). Moreover, autistic individuals often present with multiple SIB types, not a single SIB type, and this study does not examine the associated variables linked to the various combinations of SIB types an individual can present with. It is important to note that many of the variables examined here were by caregiver report, highlighting a need to expand this work using multi-method approaches, including the addition of objective measures (i.e., physiological and accelerometry data; Cantin-Garside et al., 2020; Northrup et al., 2022) and direct observation coding. Similarly, the RBS-R SIB is a caregiver report which reflects frequency, difficulty stopping, and interference to generate a severity rating, and future research may consider these aspects separately, in addition to objective measures (e.g., tissue damage). Finally, many variables included in the analysis were not significant (Supplementary Materials S2), including variables that are frequently related to self-injury in non-autistic samples (i.e., depression and anxiety symptoms, history of trauma), and it will be important for future work to better parse these potential relationships for autistic people. Given the breadth of literature on SIB and behavioral functions, it will be important for future work to examine how emotion dysregulation, psychiatric, medical, and demographic factors further relate to these functions.

## Conclusion

Each type of SIB had unique sets of associated factors in autistic youth. More repetitive motor movements were most strongly associated with SIB, followed by lower communication and daily living skills, though no one variable related to all SIB types, highlighting a need for parsing these behaviors. Several signals for future study of associations between specific types of SIB and psychiatric and emotional symptoms emerged. Self-hitting against surface/object had associations with intense emotional reactions, difficulty calming down, switching emotions, and becoming upset without a reason, and physically attacking others. ADHD symptoms had associations with rubbing/scratching and skin picking. Inserts finger/object had several medical conditions associations (e.g., headaches, allergies, pica, and high prolonged fever), raising the need to investigate medical etiologies carefully when this specific type of SIB presents. Further study of SIB types would benefit from an examination of longitudinal data to uncover causal relationships that could inform screening, prevention, and intervention for these often difficult-to-treat and highly impairing behaviors.

## Supporting information

Antezana et al. supplementary material Antezana et al. supplementary material

## Footnotes

1 Although person-first language is typically used when referring to psychiatric diagnoses, in autism research identity-first language is frequently used to be consistent with anti-ableist recommendations (e.g., Bottema-Beutel et al., 2021). This manuscript primarily uses identity-first language and adjusted terminology in describing certain scales to be consistent with such recommendations (e.g., use of ‘repetitive motor movements’ rather than ‘stereotyped behavior’). The authors acknowledge that these language preferences vary and that the best practice in everyday settings is to ask and use what individuals and families prefer.

## Supplementary material

The supplementary material for this article can be found at http://doi.org/10.1017/S0033291725000637.

## Presentation information

This study was presented as part of a panel presentation at the International Meeting for Autism Research, Melbourne, Australia, May 15–18, 2024.
