# Supplementary material for: Using machine learning to identify features associated with different types of self-injurious behaviors in autistic youth
Source: Psychol Med. 2025 Mar 31;55:e98. doi: 10.1017/S0033291725000637 (PMC12094640; doi:10.1017/S0033291725000637)
Supplement: Antezana et al. supplementary material [file S0033291725000637sup001.docx]

**Supplementary Material**

**Appendix 1.** Measures

The SCQ (Rutter et al., 2003) total score was used as a measure of lifetime autistic traits. The SCQ consists of 40 items and was developed for both speaking and non-speaking youth. Total scores range from 0 to 39 with higher scores representing more lifetime autistic traits. The SCQ has excellent internal consistency (α = 0.90) and validity with autism measures (Berument et al., 1999).

The Vineland-II (Sparrow et al., 2005) was used as a measure of adaptive behavior. Domain scores of communication, daily living, and socialization, and the adaptive behavior composite score were used in analyses. Domain scores are standardized scores based on norms with a mean of 100 and a standard deviation of 15, and the adaptive behavior composite score is a sum of the standardized scores for the three domains. Higher scores indicate more adaptive behaviors.

The ABC (Aman et al., 1985a, 1985b; Kaat et al., 2014) measures various behavioral challenges commonly observed in individuals with intellectual and developmental disorders. We utilized four ABC subscales: Lethargy, Stereotypic Behavior, Hyperactivity/Noncompliance, and Inappropriate Speech. The ABC Irritability subscale was not included in analyses due to significant overlap with the EDI Reactivity subscale (Stoddard et al., 2020). The ABC subscales have good-to-excellent internal consistency (αs = 0.85-94) and good convergent and divergent validity with behavioral difficulties (Kaat et al., 2014).

The CASI (Gadow & Sprafkin, 2010) assesses emotional and behavioral difficulties as they relate to DSM-5 diagnoses among youth. T-scores were derived from the following: ADHD-Inattentive, ADHD-Hyperactive/Impulsive, ADHD-Combined, Oppositional Defiant, Generalized Anxiety, Social Anxiety, Separation Anxiety, Schizophrenia, Major Depressive Episode, Persistent Depression, and Manic episode. Of note, social anxiety and manic episode scales were excluded due to the missing data criterion of the analytic approach. This measure has been used in samples of autistic youth, including those with low intellectual abilities (Antezana et al., 2015; Kaat et al., 2013; Rosen et al., 2019; Sukhodolsky et al., 2008), with acceptable-to-excellent internal consistency (αs = 0.78-96) and validity with interview measures of psychiatric disorders (Meyers et al., 2006).

The EDI is a 30-item caregiver report designed to measure emotion dysregulation difficulties in autistic individuals (Mazefsky, Day, et al., 2018; Mazefsky, Yu, et al., 2018). It was developed based on National Institutes of Health Patient Reported Outcomes Measurement Information System (PROMIS) guidelines. The EDI assesses two areas of emotion dysregulation: a 24-item reactivity scale, and 6-item dysphoria scale. Items were rated on a 5-point scale based on behavior during the previous 7 days, from 0 (“not at all”) to 4 (“very severe”). The EDI subscales have excellent internal consistency and validity with emotional and behavioral symptoms (Conner et al., 2021; Mazefsky, Day, et al., 2018; Mazefsky, Yu, et al., 2018; Mills et al., 2022). EDI item level scores were used in analyses to examine emotion difficulties as they relate to SIB.

**Appendix 2.** Non-significant variables.

Seventy-five variables were included in the analyses. Significant relationships were found for 42 of the variables, while 33 were included in the analyses but did not relate to any SIB type. The non-significant variables are listed below.

Demographics

Age, ethnicity (Hispanic vs. not Hispanic), and race (White vs. non-White).

Medical Conditions

History of asthma, bowel and bladder problems, ear infections, hearing loss, seizures or convulsions, tics, visual/eye problems, or trauma, family simplex or multiplex status, known genetic condition.

Psychiatric Symptoms

Generalized anxiety, major depressive episode, and persistent depression.

Emotion Dysregulation

EDI-Reactivity items were appears angry or irritable, has explosive outbursts, cries or stays angry for 5 minutes or longer, breaks down if told he/she can’t do something, seems on edge, emotions go from 0 to 100 instantly, tense or agitated and unable to relax, seems to be in a rage, reactions usually are more severe than the situation calls for, has mood swings, difficult to distract if he/she is frustrated or upset, cannot change his/her mood even with your best effort, and easily triggered/upset.

EDI-Dysphoria items were very little makes him/her happy, does not seem to enjoy anything, not responsive to praise or good things happening, and refuses to leave the house or go to school or activities unless forced.

References

Aman, M. G., Singh, N. N., Stewart, A. W., & Field, C. J. (1985a). Psychometric characteristics of the Aberrant Behavior Checklist. *American Journal of Mental Deficiency*, *89*, 492–502.

Aman, M. G., Singh, N. N., Stewart, A. W., & Field, C. J. (1985b). The aberrant behavior checklist: A behavior rating scale for the assessment of treatment effects. *American Journal of Mental Deficiency*, *89*, 485–491.

Antezana, L., Mosner, M. G., Troiani, V., & Yerys, B. E. (2015). Social-Emotional Inhibition of Return in Children with Autism Spectrum Disorder Versus Typical Development. *Journal of Autism and Developmental Disorders*. https://doi.org/10.1007/s10803-015-2661-9

Berument, S. K., Rutter, M., Lord, C., Pickles, A., & Bailey, A. (1999). Autism screening questionnaire: Diagnostic validity. *The British Journal of Psychiatry: The Journal of Mental Science*, *175*, 444–451.

Conner, C. M., Golt, J., Shaffer, R., Righi, G., Siegel, M., & Mazefsky, C. A. (2021). Emotion Dysregulation is Substantially Elevated in Autism Compared to the General Population: Impact on Psychiatric Services. *Autism Research*, *14*(1), 169–181. https://doi.org/10.1002/aur.2450

Gadow, K. D., & Sprafkin, J. (2010). *Child & Adolescent Symptom Inventory—Fourth Edition Revised*. Checkmate Plus.

Kaat, A. J., Gadow, K. D., & Lecavalier, L. (2013). Psychiatric Symptom Impairment in Children with Autism Spectrum Disorders. *Journal of Abnormal Child Psychology*, *41*(6), 959–969. https://doi.org/10.1007/s10802-013-9739-7

Kaat, A. J., Lecavalier, L., & Aman, M. G. (2014). Validity of the Aberrant Behavior Checklist in Children with Autism Spectrum Disorder. *Journal of Autism and Developmental Disorders*, *44*(5), 1103–1116. https://doi.org/10.1007/s10803-013-1970-0

Mazefsky, C. A., Day, T. N., Siegel, M., White, S. W., Yu, L., Pilkonis, P. A., & Autism and Developmental Disabilities Inpatient Research Collaborative (ADDIRC). (2018). Development of the Emotion Dysregulation Inventory: A PROMIS®ing Method for Creating Sensitive and Unbiased Questionnaires for Autism Spectrum Disorder. *Journal of Autism and Developmental Disorders*, *48*(11), 3736–3746. https://doi.org/10.1007/s10803-016-2907-1

Mazefsky, C. A., Yu, L., White, S. W., Siegel, M., & Pilkonis, P. A. (2018). The Emotion Dysregulation Inventory: Psychometric Properties and Item Response Theory Calibration in an Autism Spectrum Disorder Sample. *Autism Research : Official Journal of the International Society for Autism Research*, *11*(6), 928–941. https://doi.org/10.1002/aur.1947

Meyers, K., Hagan, T. A., McDermott, P., Webb, A., Randall, M., & Frantz, J. (2006). Factor Structure of the Comprehensive Adolescent Severity Inventory (CASI): Results of Reliability, Validity, and Generalizability Analyses. *The American Journal of Drug and Alcohol Abuse*, *32*(3), 287–310. https://doi.org/10.1080/00952990500479464

Mills, A. S., Tablon-Modica, P., Mazefksy, C. A., & Weiss, J. A. (2022). Emotion dysregulation in children with autism: A multimethod investigation of the role of child and parent factors. *Research in Autism Spectrum Disorders*, *91*, 101911. https://doi.org/10.1016/j.rasd.2021.101911

Rosen, T. E., Spaulding, C. J., Gates, J. A., & Lerner, M. D. (2019). Autism severity, co-occurring psychopathology, and intellectual functioning predict supportive school services for youth with autism spectrum disorder. *Autism*, *23*(7), 1805–1816. https://doi.org/10.1177/1362361318809690

Rutter, M., Bailey, A., & Lord, C. (2003). *The Social Communication Questionnaire Manual*. Westerm Psychological Services.

Sparrow, S. S., Cicchetti, D. V., & Balla, D. A. (2005). *Vineland Adaptive Behavior Scales, Second Edition (Vineland-II)*. NCS Pearson.

Stoddard, J., Zik, J., Mazefsky, C. A., DeChant, B., & Gabriels, R. (2020). The Internal Structure of the Aberrant Behavior Checklist Irritability Subscale: Implications for Studies of Irritability in Treatment-Seeking Youth With Autism Spectrum Disorders. *Behavior Therapy*, *51*(2), 310–319. https://doi.org/10.1016/j.beth.2019.09.006

Sukhodolsky, D. G., Scahill, L., Gadow, K. D., Arnold, L. E., Aman, M. G., McDougle, C. J., McCracken, J. T., Tierney, E., Williams White, S., Lecavalier, L., & Vitiello, B. (2008). Parent-Rated Anxiety Symptoms in Children with Pervasive Developmental Disorders: Frequency and Association with Core Autism Symptoms and Cognitive Functioning. *Journal of Abnormal Child Psychology*, *36*(1), 117–128. https://doi.org/10.1007/s10802-007-9165-9
